# Potential of chimeric antigen receptor (CAR)‐redirected immune cells in breast cancer therapies: Recent advances

**DOI:** 10.1111/jcmm.17465

**Published:** 2022-06-28

**Authors:** Marzieh Nikoo, Mohammad Rudiansyah, Dmitry Olegovich Bokov, Nurlan T. Jainakbaev, Wanich Suksatan, Mohammad Javed Ansari, Lakshmi Thangavelu, Supat Chupradit, Amir Zamani, Ali Adili, Navid Shomali, Morteza Akbari

**Affiliations:** ^1^ Department of Immunology, School of Medicine Kermanshah University of Medical Sciences Kermanshah Iran; ^2^ Division of Nephrology & Hypertension, Department of Internal Medicine, Faculty of Medicine Universitas Lambung Mangkurat / Ulin Hospital Banjarmasin Indonesia; ^3^ Institute of Pharmacy Sechenov First Moscow State Medical University Moscow Russian Federation; ^4^ Laboratory of Food Chemistry Federal Research Center of Nutrition, Biotechnology and Food Safety Moscow Russian Federation; ^5^ Kazakh‐Russian National Medical University Almaty Kazakhstan; ^6^ Faculty of Nursing, HRH Princess Chulabhorn College of Medical Science Chulabhorn Royal Academy Bangkok Thailand; ^7^ Department of Pharmaceutics, College of Pharmacy Prince Sattam Bin Abdulaziz University Al‐kharj Saudi Arabia; ^8^ Department of Pharmacology, Saveetha Dental College, Saveetha Institute of Medical and Technical Science Saveetha University Chennai India; ^9^ Department of Occupational Therapy, Faculty of Associated Medical Sciences Chiang Mai University Chiang Mai Thailand; ^10^ Shiraz Transplant Center, Abu Ali Sina Hospital Shiraz University of Medical Sciences Shiraz Iran; ^11^ Department of Oncology Tabriz University of Medical Sciences Tabriz Iran; ^12^ Senior Adult Oncology Department, Moffitt Cancer Center, University of South Florida Tampa Florida USA; ^13^ Department of Immunology Tabriz University of Medical Sciences Tabriz Iran

**Keywords:** breast cancer, chimeric antigen receptor (CAR), immunotherapy, natural killer (NK) cells, T cells

## Abstract

Despite substantial developments in conventional treatments such as surgery, chemotherapy, radiotherapy, endocrine therapy, and molecular‐targeted therapy, breast cancer remains the leading cause of cancer mortality in women. Currently, chimeric antigen receptor (CAR)–redirected immune cell therapy has emerged as an innovative immunotherapeutic approach to ameliorate survival rates of breast cancer patients by eliciting cytotoxic activity against cognate tumour‐associated antigens expressing tumour cells. As a crucial component of adaptive immunity, T cells and NK cells, as the central innate immune cells, are two types of pivotal candidates for CAR engineering in treating solid malignancies. However, the biological distinctions between NK cells‐ and T cells lead to differences in cancer immunotherapy outcomes. Likewise, optimal breast cancer removal via CAR‐redirected immune cells requires detecting safe target antigens, improving CAR structure for ideal immune cell functions, promoting CAR‐redirected immune cells filtration to the tumour microenvironment (TME), and increasing the ability of these engineered cells to persist and retain within the immunosuppressive TME. This review provides a concise overview of breast cancer pathogenesis and its hostile TME. We focus on the CAR‐T and CAR‐NK cells and discuss their significant differences. Finally, we deliver a summary based on recent advancements in the therapeutic capability of CAR‐T and CAR‐NK cells in treating breast cancer.

## 1 INTRODUCTION

Breast cancer is a heterogeneous disorder with the highest prevalence among malignancies, and it is still one of the top causes of cancer death in women around the world. Breast cancer is divided into molecular subsets with distinctive biology and clinical characteristics.^1^, ^2^ Every year, almost 2 million new breast cancer cases are identified worldwide, and over half a million individuals die from the disease due to recurrence or metastasis.^3^, ^4^, ^5^, ^6^ Early detection and significant advances in standard conventional modalities (e.g., chemotherapy, radiotherapy, surgery, and hormone therapy) have improved cure rates and quality of life in women with localized breast cancer. At the same time, some subsets with distant metastases remain the primary concern for treatment.^7^, ^8^, ^9^, ^10^ Consequently, using innovative therapeutic modalities in the treatment of breast cancer is urgently required to open up new avenues to apply novel therapeutic targets to decrease the disease's recurrence and death rates.

Cancer immunotherapy, which exploits immune cells' natural anticancer capacities, has emerged as one of the most promising options for potentiating the process of cancer cell elimination.^11^ Adoptive cell‐based antitumor therapy has become a landmark event and a rapidly developing modality for cancer‐targeted therapy in recent decades.^12^, ^13^ Dendritic cells (DCs), T cells, and alloreactive natural killer (NK) cells are some of the immune effector cells used in cancer cellular therapy.^14^ As a crucial component of adaptive immunity, T cells and NK cells, as the primary innate immune cells, have received tremendous interest in cancer therapy, mainly contributing to cancer elimination and immune surveillance.^15^, ^16^

However, genetic and epigenetic modifications in the tumour microenvironment associated with tumour cell evasion from immune responses cause antitumor immune responses to be delayed, changed, or even resistant, permitting tumour development.^17^, ^18^, ^19^, ^20^, ^21^ Meanwhile, breast cancer may evade immune surveillance and increase malignant persistence through various mechanisms. They include the recruitment of regulatory T cells and myeloid‐derived suppressor cells (MDSCs) to the TME, changes in the expression of NK cell activating receptors that affect their interaction with other cells, neutralization of T cell effectors, expressing immune checkpoints, and alteration in the capacity of myeloid dendritic cells and plasmacytoid DCs.^18^, ^22^, ^23^

Scientists have devised techniques to redirect immune effector cells and thus boost anticancer properties, concomitantly inhibiting immune escape from circumventing these mechanisms. As a result, recombinant constructs known as chimeric antigen receptors (CARs) have been used to genetically engineer T and NK immune effector cells to improve adoptive cellular therapy and tumoricidal activities.^24^, ^25^ CARs are synthetic surface receptors that have been broadly used to redirect T and NK cells and can recognize a specific target antigen on the surface of cancer cells. CARs activate redirected effector cells and eventually tumour cell lysis upon detection. The basic CAR construct comprises a single‐chain variable fragment (scFv; ectodomain) that serves as an extracellular antigen‐recognition domain and is linked to a diversity of intracellular signalling domains (endodomain).^13^, ^26^, ^27^ CAR proteins can recognize a broad range of MHC‐independent tumour antigens, allowing them to attack more tumour cells.^28^, ^29^, ^30^

Meanwhile, adoptive transfer of redirected T and NK cells expressing CAR has demonstrated empowering outcomes in treating a variety of haematological malignancies. In contrast, a comparable impact on solid tumours has not been observed.^31^, ^32^ However, detecting a relevant target antigen and using complementary genetic strategies to protect these redirected cells from immunosuppressive signals delivered within the tumour microenvironment (TME) could pave the way to use this technique in solid tumours, particularly breast cancer.^9^, ^23^

The current review first discusses the pathogenesis and function of effector cells in the breast cancer microenvironment, followed by a discussion of recent findings in CAR T cell therapy and CAR NK cell therapy in breast cancer, with a particular emphasis on last decade reports.

## 2 BREAST CANCER PATHOGENESIS

Breast cancer is a collection of multifactorial and phenotypically distinct disorders with varying genetic and histologic features that influence clinical outcome prediction and therapy selection.^33^, ^34^ Although the aetiology of breast cancer and how the normal epithelium transforms into a malignant form is uncertain, multiple risk factors such as age, reproductive factors, personal or family history of breast disease, lifestyle, genetic predisposition, and environmental factors have been frequently linked to the progression of this heterogeneous disease.^33^, ^35^, ^36^

Breast cancer family history is an essential factor of disease risk. Around 20–25% of patients have a positive family history, and only 5–10% of all breast cancers are related to gene mutations inherited from a parent.^37^, ^38^, ^39^ Breast cancer 1 (BRCA1) and breast cancer 2 (BRCA2) tumour‐suppressor genes have been identified as two significant susceptibility genes in breast cancer, with mutations in the BRCA1 and BRCA2 genes involved at least 30% of hereditary breast cancer cases.^37^, ^40^ In addition to BRCA1 and BRCA2, germline mutations in five additional susceptibility genes, including tumour protein P53 (TP53), phosphatase and tensin homologue (PTEN), checkpoint kinase 2 (CHEK2), ataxia telangiectasia mutated (ATM), and partner and localizer of BRCA2 (PALB2), have been recognized as cancer‐related genes in breast cancer patients.^41^, ^42^, ^43^, ^44^ Germline mutations in the TP53 gene cause Li‐Fraumeni syndrome, with a high chance of developing early‐onset breast cancer. Furthermore, mutations in the PTEN genes, which cause Cowden syndrome, and serine/threonine kinase 11 (STK11), which identifies as a causative gene in Peutz‐Jeghers syndrome, have been linked to an increased risk of breast cancer. Investigations have shown that pathogenic mutations in *BRCA1/BRCA2* increase the risk of BC by 10‐ to 20 fold. Besides, mutations in *TP53* also give a high chance of BC, so a mutation in the TP53 was found in 65–80% of basal or TNBC breast cancers.^45^, ^46^ According to a meta‐analysis of BRCA1 and BRCA2 carrier families, the lifetime risk of breast cancer varies from 65% to 81% for BRCA1 and 45% to 85% for BRCA2. Another genetic variation associated with intermediate dangers of breast cancer and a 20%–40% lifetime chance of getting breast cancer includes the CHEK2, ATM, and PALB2 genes involved in the DNA repair process.^33^ Analysing mutations with correct and reliable genetic testing in the significant genes (BRCA1 and BRCA2) and less commonly mutated genes (such as PTEN) and subsequent genetic counselling in high‐risk women can be beneficial in the early detection and/or prevention of breast cancer development (Table 1).

**TABLE 1:** Breast cancer susceptibility mutated genes

Mutated genes	Inherited cancer syndromes	Proportion of the familial component of breast cancer (%)	Lifetime risk in women % (relative risks)	Ref
BRCA1	Hereditary breast and ovarian cancer	5–10	60–85	45,212,213
BRCA2	Hereditary breast and ovarian cancer	5–10	40–85	
TP53	Li‐Fraumeni cancer syndrome	0.1	80–90	
PTEN	Cowden syndrome	0.02	25–50	
STK11	Peutz‐Jegher syndrome	0.04	50	
CHEK2	CHEK2‐related breast cancer	2	18–20 (3>)	
ATM	Ataxia‐telengiectasia	2	20 (3>)	
PALB2	PALB2‐related breast cancer	0.4	20 (3>)	
BRIP1	BRIP1‐related breast cancer	0.4	20 (3>)	

There are several kinds of breast cancer, each defined by unique units of the breast and specific cells that are affected. Most breast cancers result from an oncogenic transformation of the epithelial compartment of breast tissue (carcinoma), which comprises cells that line functional units of lobules and terminal mammary ducts. Sarcomas, such as phyllode tumours and angiosarcomas, are a small subset of breast cancer (1% of primary breast cancer) that arise from alteration of the connective tissue compartment of breast tissue, which consists of myofibroblasts and blood vessel cells.^47^, ^48^ Breast carcinoma, the most common type of breast cancer, progresses through three major stages: non‐invasive (or in situ), invasive, and metastatic. Non‐invasive or pre‐invasive treatment is limited to the epithelium component of pre‐existing normal ducts. Because this stage has a high potential for progression to invasive carcinoma, early detection and prompt and proper therapy are paramount in preventing progression to the invasive form. Invasive carcinoma has broken through and infiltrated the epithelial components of the breast lobules and ducts, migrating into the surrounding breast connective tissue. Although it is possible to eradicate invasive carcinoma from its primary origin of development, invasive breast cancer has the potential to spread to other organs of the body, such as lymph nodes and/or distant organs such as the lung, liver, bone, and brain, and progress to metastatic breast cancer. The risk of breast carcinoma metastasizing is not easily detected, and around 30% of women with primary‐stage breast carcinoma will progress to the metastatic stage of the disease.^33^, ^47^, ^49^

Recent advances in gene expression profiling techniques have significantly influenced our understanding of breast cancer biology.^50^, ^51^ Gene expression studies have highlighted many different molecular breast cancer subtypes related to breast cancer biology and demonstrate considerable variations in their incidence, risk factors, prognosis, and therapeutic responses.^52^ The distinction between molecular/intrinsic subtypes of breast cancer is based on a diversity of inherent genes, including hormone‐related genes, human epidermal growth factor receptor 2 (HER2)–related genes, proliferation‐related genes, and the basal cluster of genes.^44^, ^53^, ^54^ Breast tumours are classified into five molecular/intrinsic subtypes based on gene expression patterns of this cluster of genes (e.g., Luminal‐A, Luminal‐B, HER2‐enriched, basal‐like, and normal breast‐like).^55^, ^56^ Luminal A breast cancer accounts for around 40% of all breast carcinomas, whereas luminal B represents 20% of all these diseases. These luminal tumours express hormone receptors [oestrogen‐receptor (ER) and/or progesterone receptor (PR) positive] and are lower grade than HER2 subtypes, which represent HER2 gene products. Luminal‐A cancers have a better prognosis than luminal‐B cancers since they frequently have greater hormone receptor expression levels and a lower proliferation index. Luminal B tumours can be HER2‐positive and have higher levels of Ki‐67 (as proliferation‐related genes). Hormonal therapy treats both luminal malignancies.^33^, ^55^ HER2‐enriched breast cancer accounts for 10% to 15% of all breast carcinomas and is associated with negative ER and PR expression and increased levels of HER2 and proliferation‐related gene expression.^57^ HER2‐enriched tumours have good prognosis and a greater proliferation rate than luminal subtypes, but they respond well to HER2‐targeted treatments. Triple‐negative/basal‐like breast cancer (TNBC) is distinguished by the absence of oestrogen and progesterone receptors and the expression of the HER2 genes.^58^ Making for roughly 15–20% of all invasive breast cancers, they have a more aggressive phenotype and a greater recurrence rate than other subtypes. Besides, BRCA1‐associated breast tumours are more likely to exhibit a basal‐like phenotype.^59^ Furthermore, TNBC was found in a high proportion of women under the age of 40 and African–American women.

## 3 BREAST CANCER TREATMENT

There are currently no benefit treatment options for TNBC patients and approved targeted therapies are ineffective in these patients. Typical breast cancer has similar characteristics to luminal A disorder, but its prognosis is somewhat worse than that of the luminal A subtype.^33^, ^60^, ^61^, ^62^ Chemotherapy, immunotherapy, radiation, and targeted therapy, among other options, are available to patients with breast cancer. A combination of two or three treatment approaches is most often applied to treat breast cancer patients. In addition, hormone receptors on malignant cells can be used as therapeutic targets to inhibit downstream survival pathways and limit tumour development. However, the outcomes of these treatments differ in various subtypes, and in many cases, genetic and epigenetic alterations in the tumour microenvironment and escape mechanisms in breast cancer cells lead to evasion of the cytotoxic activity of therapeutic regimes, as well as immune surveillance, resulting in recurrence and metastasis.^61^, ^63^, ^64^

## 4 IMMUNE CELL DYSFUNCTIONS IN BREAST CANCER

The developing breast cancer microenvironment comprises biological components such as increasing malignant cells, immune cells, adipocytes, fibroblasts, blood vessels, and tumour stroma components such as growth factors and cytokines, chemokines, prostaglandins (PGs), and others. The innate and adaptive immune systems are essential components that play a dual role in breast carcinogenesis.^65^, ^66^, ^67^ During tumour progression and the transition from the early stages of tumorigenesis to the developed and metastatic phenotype, genetic instability and the accumulation of molecular changes in tumour cells, combined with changes in normal tissue homeostasis, result in immune evasion and decreased expression of all phases of immune surveillance. The heterogeneous TME contains a variety of accumulated suppressive cells and soluble suppressive factors that contribute to modifying and impairing the function of immune antitumor effector cells, causing tumour progression.^68^, ^69^

### 4.1 T cell dysfunctions in breast cancer

T cells, which comprise naïve, memory, effector, and regulatory T cells (Treg), infiltrate the heterogeneous TME as significant components of adaptive immunity.^70^ Most lymphocytes invading the tumour may be seen in TNBC and HER2‐positive malignancies, but these cells are less prevalent in luminal type breast cancers, with the least number in luminal A‐type.^18^, ^68^ According to a previous study, once tumour‐specific T cells meet the tumour‐specific antigen early after tumour initiation, they enter a state of dysfunctional exhaustion. Dysfunctional CD8 + T cells are defined by changes in several intrinsic transcriptional and metabolic factors, as well as upregulation of immune checkpoints and multiple co‐inhibitory receptors, such as cytotoxic T lymphocyte–associated antigen‐4 (CTLA‐4), programmed cell death protein 1 (PD‐1), T cell immunoreceptor with Ig and ITIM domains (TIGIT), lymphocyte activating 3 (LAG3), and T cell immunoglobulin and mucin domain–containing protein 3 (TIM3). Such molecules have been associated with failure in effector functions such as cytotoxicity and proliferation.^71^, ^72^, ^73^ Apart from T cell self‐regulation, the TME contains a variety of immunosuppressive cells that contribute to T cell dysfunction.^74^ Treg cells, the most common type of CD4+ T cell, exhibit a high level of the transcription factor forkhead box P3 (FOXP3). They recruit and expand via ICOS–ICOSL interaction in the microenvironment of primary breast cancer and disrupt immuno‐surveillance. These cells suppress effector T cell activation, proliferation, and survival by upregulating inhibitory receptors or secreting immunosuppressive molecules such as transforming growth factor‐β (TGF‐β) and interleukin‐10 (IL‐10).^75^, ^76^, ^77^

Macrophages in tumours predominantly have an M2‐like phenotype and contribute to immune suppression and T cell dysfunction by releasing a variety of inhibitory cytokines and factors such as IL‐10, TGF‐β, and reactive oxygen species (ROS), as well as amino acid–degrading enzymes such as arginase 1 (Arg‐1) and indoleamine‐2,3‐dioxygenase (IDO). Tumour‐associated macrophages (TAMs) also may promote the overexpression of PD‐L1 in monocytes, which enables T cell exhaustion following binding to PD‐1 on the surface of CD8+ T cells.^68^, ^78^, ^79^, ^80^

Myeloid‐derived suppressor cells (MDSCs) are a diverse population of immature myeloid cells observed in the TME. Due to various mechanisms, they inhibit CD4+ and CD8+ T cell function, including high levels of Arg‐1 and inducible nitric oxide synthase (iNOS) expression, either separately or in synergism. They prevent CD8+ T cells from responding to antigens, reduce CD3z‐chain biosynthesis, and increase nitric oxide and reactive oxygen species release, which impede T cell growth and differentiation and eventually induce T cell exhaustion. Furthermore, elevated IDO expression in MDSCs in breast cancer mediates immunosuppressive effects on T cells by reducing CD8+ T cell proliferation and blocking interferon‐gamma (IFN‐γ) production.^81^, ^82^, ^83^, ^84^

Cancer‐associated fibroblasts (CAFs) are another immunosuppressive cell found in the TME that generate TGF‐β and vascular endothelial growth factor (VEGF) and suppress T cell activity. Moreover, studies have demonstrated that removing CAFs from the TME in breast cancer decreases the recruitment of TAMs, MDSCs, and T regulatory cells and reduces tumour angiogenesis and lymphangiogenesis.^85^, ^86^, ^87^ Given that T cells play a pivotal role in the adaptive immune system, there is an urgent need to boost and sustain anticancer responses as tumours progress. Genetically modified T cells, such as CAR engineered T‐cells, have emerged as a novel avenue in treating tumour‐induced T cell dysfunction and have offered promising evidence in many tumour types. Abundant evidence highlights the success of CAR T cell therapy in the treatment of patients with haematological disorders.^88^, ^89^, ^90^

### 4.2 NK cell dysfunctions in breast cancer

NK cells are a fundamental part of innate immunity, capable of recognizing and killing malignant cells by cytotoxicity and cytokine release without previous activation.^91^, ^92^ Hypoxia, low pH, and low nutritional contents in the TME have been associated with tumour progression. The accumulation of soluble mediators released by immunosuppressive cells has been implicated in impaired NK cell function.^93^, ^94^ NK cell functions are profoundly altered in the TME, with decreased NK cell infiltration in tumours, increased death of NK cells, impaired metabolism and maturation, and reduced NK cell activity, all associated with significant phenotypic changes.^95^

Circulating NK cells and tumour‐infiltrating NK cells isolated from noninvasive and invasive breast cancer patients show reduced expression of the significant effector factors, such as IFN‐γ, CD107a, granzyme B, Fas ligand, tumour necrosis factor–related apoptosis‐inducing ligand (TRAIL), and perforin.^96^ In addition, the expression of activating NK cell receptors (such as NKp30, NKG2D, DNAM‐1, and CD16) decreased while inhibitory receptors (e.g., NKG2A) increased, related to impaired NK cell activity in breast cancer.^96^ It has been reported that the elevated local concentrations of various stromal‐derived factors such as IL‐10 and TGF‐β serves critical roles in tumour‐mediated disruption of normal NK cell function. Furthermore, L‐kynurenine, an IDO1‐derived metabolite, inhibits NK cell proliferation and cytotoxicity while also decreasing the expression of NKp46 and NKG2D.^93^, ^94^, ^97^ As a result, developing strategies to boost NK cell antitumor activity and restore NK cell cytotoxicity is crucial. Given the success of genetically altered therapeutic T‐cells, particularly CAR T‐cells, in cancer treatment, genetic modification of NK cells to produce a more effective NK cell–based cancer immunotherapy seems appropriate.^16^, ^32^

## 5 IMMUNE CELL SOURCES FOR CAR‐BASED TARGETED THERAPY IN BREAST CANCER

In recent years, genetically modifying immune cells to express CARs has represented a novel adoptive cell therapy strategy in treating various progressive cancers. The genetic modification of functional T and NK cells depends on efficient and permanent gene transfer.^15^, ^24^, ^32^ Immune cells can be acquired from numerous cell sources using leukapheresis, discussed further below.

### 5.1 T cells

Functional T cells are isolated from a patient's own peripheral blood mononuclear cells as an autologous source of T cells for CAR‐T development in breast cancer. Although autologous CAR T treatment has given rise to excellent outcomes, this platform's expensive and time‐consuming nature remains the primary issue, particularly for patients with highly proliferative diseases. Moreover, autologous T cells may be ineffective in treating various cancers due to immunosuppressive processes generated by the tumour microenvironment in breast cancer.^98^, ^99^

T cells can also be obtained from healthy donors and allogeneic CAR‐T cells. This principle simplifies and standardizes CAR‐T manufacturing based on donor selection and processing. Furthermore, allogeneic cell manufacturing produces batches of products that are instantly available. Allogeneic T cells for CAR‐T cell manufacturing are typically derived from peripheral blood mononuclear cells (PBMCs) and, in rare cases, stem cells such as umbilical cord blood (UCB), induced pluripotent stem cells (iPSCs), or embryonic stem cells (ESCs).^99^

Peripheral blood T cells obtained from healthy donors are significant because of their quick and easy availability and the ability to manufacture many vials from a single apheresis product. Furthermore, on this platform, a bank of heterogeneous cells with different subtypes of human leukocyte antigen (HLA) complex may be established, from which batches that match the patient's HLA type can be selected.^99^ Allogeneic T cell transplantation may increase the risk of severe graft‐versus‐host disease (GVHD) due to significant HLA differences between the donor and recipient or minor histocompatibility of antigens with genetic polymorphism of cytokines.^100^, ^101^

Furthermore, T cells can be derived from UCB with no or little risk of GVHD, which is associated with a distinct antigen‐naive status and malfunction in nuclear factor of activated T cells (NFAT) signalling and diminished reactivity.^102^, ^103^ Furthermore, iPSCs can provide an unlimited and homogenous T cell supply for CAR‐T cell production, which can be banked and used indefinitely. It should be noted that the presence of endogenous TCR or HLA mismatch limits the platform's applicability in breast cancer. Meanwhile, genome editing technology may remove endogenous TCR and HLAs from iPSCs and create off‐the‐shelf iPSC‐derived CAR‐T cells.^98^, ^104^, ^105^ Despite this, clinical studies have yet to study the platform's safety and efficacy.

### 5.2 NK cells

Adoptive cell immunotherapy functional NK cells can be obtained from autologous or allogeneic sources. Patient PBMCs or stem cells can be used to isolate or generate autologous NK cells. Because NK cells account for roughly 10%–15%of blood lymphocytes, they should be increased ex vivo. Cytokine therapy or co‐culture with a feeder cell line.^106^ NK cells generated from stem cells are easy to store and purify; yet, variations in functional receptor expression and cytotoxicity effects have been identified compared to NK cells originating from the peripheral sources. Besides, self‐HLA molecules on tumour cells are recognized by adoptively transplanted autologous NK cells, which inhibit their cytotoxic activities.^106^, ^107^, ^108^ As a result, allogeneic NK cells are more effective as alternative therapeutic targets for adoptive cell immunotherapy.^32^, ^106^

Allogeneic NK cells may be produced from various sources, including PB,^26^ different NK cell lines,^109^ and stem cell–derived NK cells like UCB,^110^ ESCs,^111^ and iPSCs.^112^ Like T cells, PBMCs are the most common source of NK cells, which may be collected by lymphocyte apheresis from healthy donors. After activation, PB‐derived NK cells express various activating and inhibitory receptors, including CD16, NKG2D, the NCRs (NKp44 and NKp46), and KIRs, which have substantial destructive potential against abnormal cells.^113^ However, due to the low proportion of NK cells in peripheral blood, their collection is time‐consuming and expensive, necessitating adopting methods to expand and improve their cytotoxicity activity.^114^, ^115^ UCB‐NK cells are another source of allogeneic NK cells that can be provided more easily due to their quick availability and relative ease of collection, substantial proliferative competency, and low risk of GVHD.^116^, ^117^ In contrast to PB‐NK cells, UCB‐NK cells are naturally immature and exhibit a reduced rate of activating receptors such as NKp46, NKG2C, IL‐2R, DNAM‐1, CD57; adhesion molecules such as CD2, CD11a, CD18, CD62L, and CD16; as well as a higher rate of inhibitory receptor NKG2A.^118^, ^119^ Thus, techniques such as exposing them to stimulatory cytokines or co‐cultivating them with feeder cell lines help their maturation and boost their ex vivo expansion and activation.^120^

CD34‐positive cells, such as iPSCs, provide an additional source of allogeneic NK cells with a high proliferating capacity for creating many homogenous NK cells with high cytotoxic potential.^121^, ^122^ These cells, like PB‐NK cells, exhibit normal levels of activating NK receptors, including NKG2D, NKp46, Fas, and TRAIL, but a lower amount of killer inhibitory receptors than PB‐NK cells.^123^ Similarly, iPS‐NK exhibits the same PB‐NK characteristics in response to the tumour microenvironment, including expansion, persistence, and cytotoxicity. Furthermore, iPS‐NK is unexpectedly amenable to genetic engineering, constructing CAR‐ iPS‐NK with desirable characteristics such as superior durability and target specificity, resistance to exhaustion, and the ability to activate other immune cells to increase tumour inhibition.^124^

Several clonal NK cell lines, including NK‐92, NK‐YS, NKL, NKG, KHYG‐1, and others, are candidates for allogeneic CAR‐NK cell immunotherapy since they can be easily expanded to produce a more homogenous population of cells under normal circumstances.^125^ NK‐92 cells are the most studied and have consistent antitumor properties. NK‐92 cells generate more perforin, granzyme, and other cytotoxic cytokines than PB‐NK cells. However, they do not exhibit several activating receptors, such as NKp44, NKp46, and CD16, and the inhibitory receptor of KIRs, thus compromising their cytotoxic capacity.^126^, ^127^, ^128^ Furthermore, to avoid permanent allogeneic tumour engraftment and safety issues, the NK‐92 cell line must be irradiated before being injected into patients, negatively influencing their persistence in the host.^129^

## 6 THE IMPORTANCE OF ANTIGEN SELECTION

The first step in developing effective CAR therapies for breast cancer is to select appropriate target antigens. Choosing the right CAR target while considering factors such as molecules involved in tumorigenesis and tumour invasion and molecules with stable expression, high coverage, and specificity might result in an efficient and safe treatment.^24^ Tumour‐associated antigens (TAA), tumour‐specific antigens (TSA), NKG2D ligands, and stromal cell markers are typical CAR targets for CAR‐T and CAR‐NK therapy for breast cancer. TAA are surface antigens highly overexpressed on malignant cells in most cancers but should not be expressed or expressed at a low level in healthy tissues.^130^ TSA is another target antigen exclusively expressed in tumour cells and is established due to somatic mutations and gene rearrangement in these tumorous cells. Another highly expressed target in tumour‐associated endothelium or fibroblast cells is stromal cell markers, where targeting them might inhibit tumorigenesis by affecting angiogenesis or stromal development.^131^ Many studies have looked at the HER family, mesothelin (Meso), folate receptor alpha (FR‐α), NKG2D ligands, c‐MET, and mucin 1 (Muc1) as potential targets for breast cancer in vitro and in animal models that will be discussed in the following sections.^9^

## 7 OVERVIEW OF CAR STRUCTURE

**FIGURE 1:**
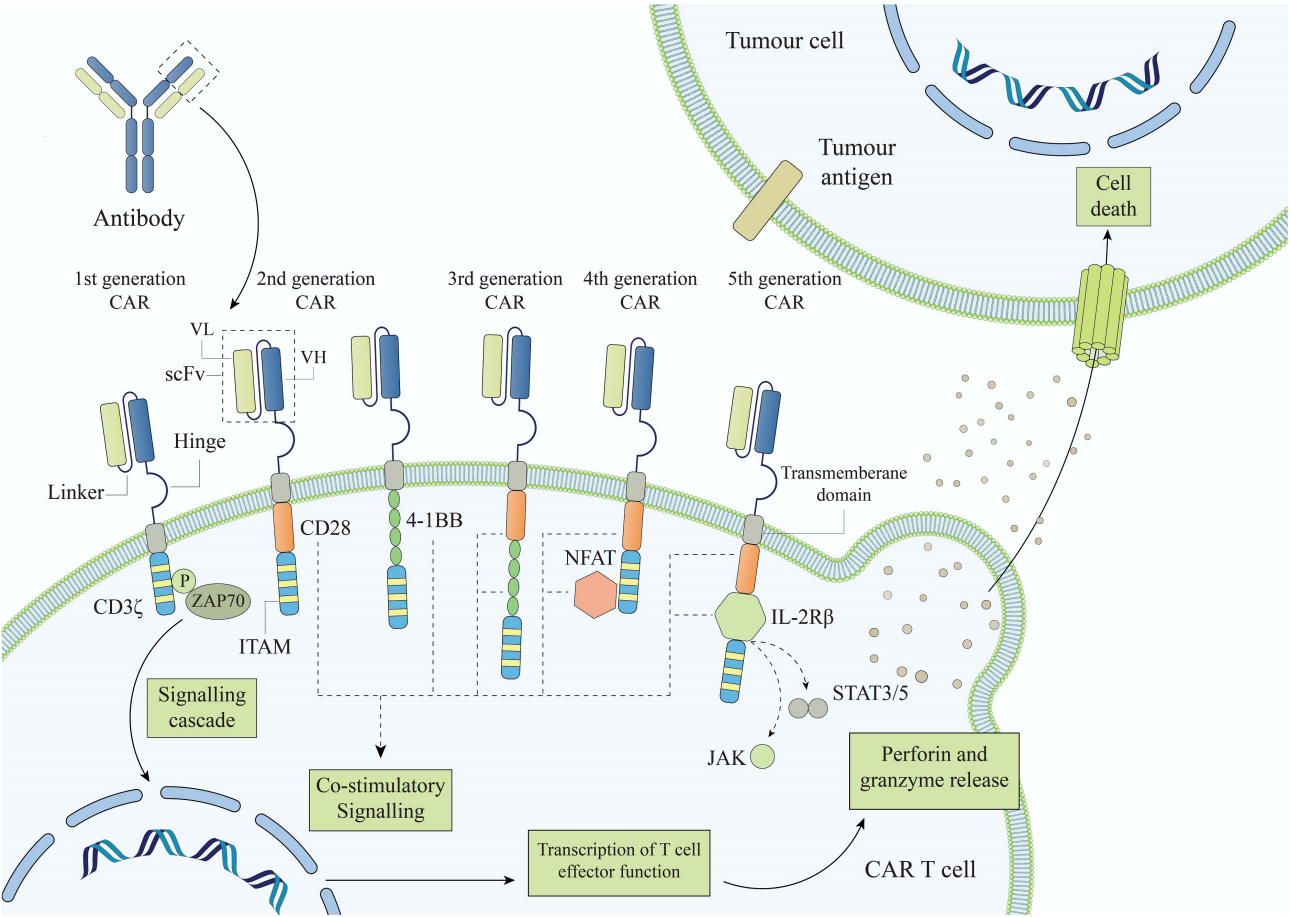
Main structure of current generations of chimeric antigen receptors (CAR). The CAR protein comprises the extracellular domain, monoclonal antibody‐derived scfv, recognizing a TAA on tumour cells independent of MHC. transmembrane domain link the recognition unit with the intracellular signalling domains. First‐generation CARs are signalled by an intracellular T‐cell activating domain, commonly the CD3 zeta chain. In the second generation of CARs, co‐stimulatory molecules, such as CD28 or 4‐1BB, were added to the transmembrane unit to heighten T cell activation. Third‐generation CARs consist of two co‐stimulatory signalling molecules in tandem with the CD3ζ chain. Fourth‐generation CARs incorporate NFAT transcription factors to promote co‐stimulatory signalling and expression of a cassette containing IL‐12 genes required for T cell activation. In the fifth generation of CARs, a JAK–STAT activation domain derived from IL‐2Rβ was added to the second generation of CARs that motivates cell activation, more persistence, and averts terminal differentiation

CARs are functional synthetic proteins expressed on the surface of immune cells that generally combine the unique tumour‐identification capacity of monoclonal antibody variable regions with considerable cytotoxic and proliferative capabilities of T or NK cells.^132^, ^133^ A typical CAR comprises an extracellular antigen recognition domain, a single‐chain antibody variable fragment (scFv) that detects particular antigens in tumours and transmembrane and intracellular signalling domains.^134^, ^135^ The intracellular domains are generated from immunoreceptor tyrosine‐based activation motifs (ITAMs) found in the cytoplasmic domains of TCRs or other activating receptors. The first generation of CARs in both CAR‐T and CAR‐NK contained only CD3 as a single activation intracellular signalling domain, which was inefficient in activating immune cells and eradicating tumours. Following that, second‐ and third‐generation CARs with T cell co‐stimulatory signalling domains such as CD28, 4‐1BB (CD137), ICOS, or OX40 (CD134), in addition to CD3, were evolved to dramatically increase cytotoxicity and proliferative activity, as well as their in vivo persistence (Figure 1).^136^, ^137^, ^138^ The fourth generation can use nuclear factor of activated T cell (NFAT) to inspire a promoter related to a cassette containing IL‐12 genes.^138^ In addition, the fifth‐generation CARs are established regarding the second generation of CARs, with the addition of a JAK–STAT activation domain derived from IL‐2Rβ. This domain motivates cell expansion, averts terminal differentiation, and elicits more appropriate persistence.^139^

While 1BB‐comprising CARs could activate both T and NK cells, CD28‐comprising CARs in NK cells are less clear as one of the most costimulatory domains used in CAR‐T cells.^140^ Consequently, several researchers developed CAR structures based on the activating properties of the NK cell. For example, DNAX‐activation protein 12 (DAP12) is a transmembrane protein that signals the NK activating receptors NKG2C, NKp44, and KIR. CARs containing the signalling domain DAP12 demonstrated superior anticancer potential in primary NK cells or NK92 cell lines compared to NK cells expressing a CD3‐based CAR.^141^, ^142^ Moreover, using 2B4 (CD244) as a pivotal NK‐specific costimulatory domain in a CAR‐NK structure resulted in rapid proliferation, enhanced specific cytotoxicity, and a robust anticancer function.^143^

Recently, the fourth generation of CARs incorporates a transgene payload, such as a cytokine cassette, which enhances the cell's function by integrating the CAR and its survival environment.^144^, ^145^, ^146^ To date, tremendous efforts have been made to develop the efficacy of CAR‐redirected immune cell therapy in solid tumours such as breast cancer, including selecting appropriate targets and designing other next‐generation CAR‐redirected immune cells to restore immune cell responses in the suppressive TME along with developing novel strategies to bypass restrictions in tumour immune cell trafficking.

## 8 DIFFERENCES BETWEEN CAR‐NK AND CAR‐T CELLS

Many investigations have been developed and carried out based on the role of CAR‐NK and CAR‐T cells in the treatment of solid malignancies. Meanwhile, the biological distinctions between NK cells and T cells impact the design processes and result in variations in the outcomes of utilizing CAR‐NK cells and CAR‐T cells in cancer immunotherapy.^147^, ^148^ Various studies have focused on optimizing CAR structure to enhance the ability and activity of the CAR‐T and CAR‐NK cells.^127^, ^149^ Despite the excellent efficacy and extended duration of remission following CAR‐T cell therapy in patients with haematological malignancies, its large‐scale clinical application is limited by some drawbacks such as individual preparation and harmful effects such as cytokine release storm (CRS), neurotoxicity, and on‐target/off‐tumour effects. In this respect, the safety and feasibility of CAR‐NK cell–based immunotherapy have been demonstrated in a variety of clinical settings.^150^

### 8.1 Sources and persistence

Allogeneic NK cells have a lower or no risk of GvHD than allogeneic T cells, allowing CAR‐NK cells to be derived from various sources, including PBMCs, UCB, pluripotent stem cells, and cell lines such as NK‐92.^151^ Moreover, CAR‐NK cells have a short life cycle in the blood, which reduces cellular memory responses and the potential of on‐target/off‐target damage to normal tissues. However, CAR‐NK cells' limited in vivo persistence fences their ability to migrate and penetrate solid tumours compared with CAR‐T cells. Notwithstanding, local or intra‐peritoneal injection of CAR‐NK cells and administration of exogenous cytokines could support their survival and in vivo proliferation.^152^, ^153^, ^154^ On the other hand, exogenous cytokines have adverse effects and can increase immunosuppressive immune subsets such as Tregs. Thus, a novel strategy is to design NK cells with cytokine transgenes that secrete or express cytokines on the membrane regularly, supporting cytokine supply while minimizing its adverse effects.^155^, ^156^, ^157^

### 8.2 Cytokine content and cytotoxicity mechanism

**FIGURE 2:**
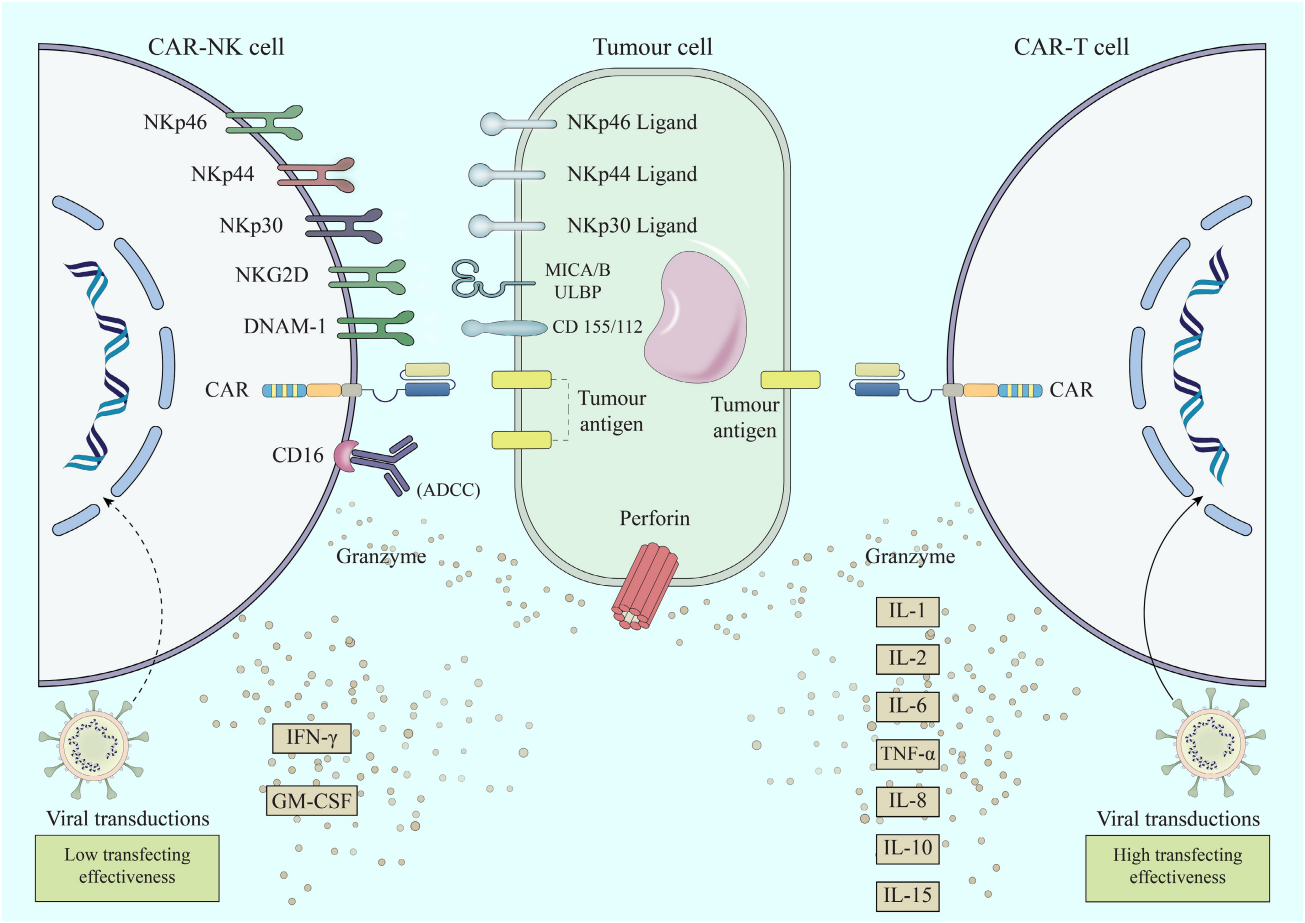
Main differences between chimeric antigen receptor (CAR)–engineered T (CAR‐T) and natural killer (CAR‐NK) cells. The ability of CAR‐NK cells in eradicating malignant cells has been related to the function of CAR structure to recognize tumour‐specific antigens along with a variety of their native activating receptors, which are not antigen‐specific and have a natural ability to detect stress‐stimulated ligands on tumour cells, including natural cytotoxicity receptors (NKp46, NKp44, and NKp30), natural killer group 2 member D (NKG2D), and DNAX accessory molecule‐1 (DNAM‐1or CD226). Moreover, the cytokine content of activated NK cells differs from that of T cells; CAR‐T cells typically secrete pro‐inflammatory cytokines such as IL‐1, IL‐2, IL‐6, TNF‐, IL‐8, IL‐10, and IL‐15, which in turn, may cause a profound adverse effect. On the other hand, the efficacy of gene transduction to CAR‐T cells differs from that of CAR‐NK cells. Although viral transductions are now the favoured technique for CAR modification of T cells, they result in lower transfecting effectiveness in NK cells

Furthermore, the cytokine content of activated NK cells differs from that of T cells; activated NK cells mainly secrete IFNγ and granulocyte‐macrophage colony‐stimulating factor (GM‐CSF), whereas CAR‐T cells typically secrete pro‐inflammatory cytokines such as IL‐1, IL‐2, IL‐6, TNF‐, IL‐8, IL‐10, and IL‐15, which in turn, facilitate CRS and severe neurologic‐related toxicities (Figure 2).^158^, ^159^ Unlike CAR‐T cells, which only inhibit tumour cells by detecting tumour‐specific antigens (TAAs) and CAR‐related mechanisms, CAR‐NK cells can also spontaneously eradicate malignant cells through a variety of their native activating receptors, including natural cytotoxicity receptors (NKp46, NKp44, and NKp30), natural killer group 2 member D (NKG2D), and DNAM‐1or CD226, and their innate ability to detect stress‐stimulated ligands on tumour cells.^32^ These receptors generally recognize stress‐stimulated ligands after the initial interaction with immune cells or long‐term therapy throughout tumour development. As a result, CAR‐modified NK could efficiently eradicate extremely diverse malignant cells lacking CAR‐targeted particular antigen through CAR‐related and NK cell receptor–dependent mechanisms.^160^, ^161^, ^162^

### 8.3 Efficacy of gene transduction

Moreover, the efficacy of gene transduction to CAR‐T cells differs from that of CAR‐NK cells. Although viral transductions are now a preferred technique for CAR modification of T cells, they have lower transfecting effectiveness in NK cells. Retroviral transfection increases the possibility of insertional mutagenesis, but lentiviral transduction has a lesser potential to introduce transgene into primary NK cells. Consequently, non‐viral vectors can circumvent these limitations and are considered a safe and cost‐effective alternative. However, the suitability of all non‐viral vectors for CAR‐NK constructs should be investigated.^26^, ^163^

## 9 OVERVIEW OF RECENT STUDIES BASED ON CAR‐T CELLS IN BREAST CANCER

The amount and quality of research in breast cancer immunotherapy directed at CAR T cells has dramatically risen in recent years.^164^ Substantial efforts are being made to improve the efficacy of CAR T therapy against solid tumours, including the identification of appropriate target antigens, evolvement of the next‐generation CAR T cells with improved capabilities, increasing the efficiency of T cell responses to moderate T cell dysfunction in the suppressive TME, and developing new strategies to overcome restrictions in tumour T cell trafficking.^165^, ^166^, ^167^ As previously mentioned, CAR T cells identify several forms of target antigens, whose proteins are among the most antigenic targets changed or overexpressed on the surface of malignant cells. Carbohydrates and glycolipids are two more CAR T‐cell targets often modified in tumour cells^168^, ^169^ (Table 2).

**TABLE 2:** Overview of different antigen‐specific CAR T‐cells preclinical studies for breast cancer

Target Ag	CAR design	CAR transduction	Key preclinical outcomes	Ref
Folate receptor‐α	CD28/4‐1BB/CD27/ CD3ζ	Lentiviral	Specific cytotoxicity of FRα‐CAR T cells when cocultured with FR‐expressing MDA‐MB‐231 BC cell line (in vitro)	^172^
Folate receptor‐α	CD27/ CD3ζ	Lentiviral	Potent cytotoxicity of FRα‐CAR T cells against FR‐expressing TNBC cell lines (in vitro) and significant tumour regression following infusion into a murine xenografts	^171^
MUC1	CD28/OX40/ CD3ζ	Retroviral	Promoted proliferation and proinflammatory cytokine production in MUC1‐CAR T cells upon exposure to MUC1 and death of MUC1(+) tumour cells (in vitro). MUC1‐CAR T cells delayed tumour growth in a xenografts	^174^
MUC1	4/7ICR 41BB/CD3ζ	Retroviral	Prolonged cytotoxic activity in MUC1+ MDA MB 468 cell lines (in vitro) and IL4‐producing MDA MB 468 tumour‐bearing animals (in vivo)	^164^
c‐Met	‐	Electroporation of mRNA	Effective cytotoxic activity of c‐Met CAR T cells against breast cancer cell lines (in vitro) and c‐Met expressing tumour xenografts in mice (in vivo)	^175^
HER2	CD28/4‐1BB/ CD3ζ	Lentiviral	Antitumour impact in xenograft mouse models of breast metastatic brain tumours (in vivo)	^178^
HER2	CD28/CD3‐ζ	Lentiviral	Induced apoptosis in the ERBB2 overexpressing human breast cancer cell line (in vitro)	^179^
EGFR	CD28/CD3ζ	Retroviral	Targeted elimination of breast cancer cell lines with a wide range of EGFR receptor profiles (in vitro) and substantial anticancer efficacy in mice with established xenografts (in vivo)	^176^
HER3/HER4	41BB/CD3ζ	Lentiviral	Boosted the killing potential of CAR‐T cells against HER3‐overexpressing SK‐BR‐3 and BT‐474 breast cancer cell lines (in vitro) and strong antitumour activity in a xenograft model with SK‐BR‐3 tumour cells (in vivo)	^180^
EGFR	CD28/CD3ζ	Lentiviral	Inhibited growth of high‐EGFR‐expressing TNBC cell lines (in vitro) and patient‐derived xenograft TNBC tumours in mice (invivo).	^181^
EGFR	CD28/4‐1BB/ CD3ζ	Lentiviral	Potent and specific suppression of EGFR‐expressing TNBC cells (MDA‐MB‐231 and MDA‐MB‐468 cell lines) (in vitro) and significant anticancer potential in a xenograft mice model (in vivo)	^182^
HERV‐ K	‐	Lentiviral	The proliferation of BC cell lines was suppressed and cytokine release was increased in the culture medium of BC cells treated with K‐CARs (in vitro) tumour development and spread to other organs successfully suppressed in a xenograft model of MDA‐MB‐231 or MDA‐MB‐435, K‐CAR T cells (in vivo)	^183^
TEM8	CD28.41BB.CD3ζ	Retroviral	Effective killing activity of TEM8+ TNBC tumour cell lines such as Hs578T, MDA‐MB‐231, MDA‐MB‐436, and MDA‐MB‐468 (in vitro). tumour regression in MDAMB‐468 tumour‐bearing mice (in vivo).	^187^
GD2	41BB/CD3ζ	Lentiviral	GD2‐CAR T cells significantly lysed GD2‐positive breast cancer cells (in vitro) as well as halted local tumour progression and completely prevented lung metastatic in an orthotopic xenograft model of TNBC (MDA‐MB‐231)(in vivo)	^188^
AXL	CD28/41BB/CD3ζ	Lentiviral	Significant cytolytic activity and cytokine secretion against AXL positive cells MDA‐MB‐231 (in vitro)inhibition of tumour development in the mouse model of TNBC MDA‐MB‐231 xenografts (in vivo)	^189^
Mesothelin	CD28/41BB/CD3ζ	Lentiviral	Strong cytotoxicity in breast cancer MDA‐MB‐231‐Luc and MCF‐7‐Luc cell lines by releasing cytokines, perforin, and granzyme B (in vitro). inhibition of tumour growth at a late stage in mice bearing MDA‐MB‐231 TNBC xenografts (in vivo).	^191^
NKG2D	CD27/41BB/CD3ζ	Lentiviral	Marked antitumor effect against TNBC MDA‐MB‐231and MDA‐MB‐468 cell lines (in vitro) and (in vivo) in MDA‐MB‐231 xenograft mice	^190^

*Note*: Many studies have been carried out that the use of different appropriate antigens, designing efficient CAR structure, and improving gene transduction methods to enhance the efficiency of CAR T therapy in breast cancer, which are summarized in the table.

Folate receptor‐alpha (FR) is a glycosylphosphatidylinositol (GPI)‐linked surface protein highly overexpressed in non‐mucinous epithelial malignancies such as ovarian, breast, and lung tumours. FR has been overexpressed in specific malignancies, such as ER‐negative, stage IV metastatic TNBC, at roughly 86%. However, its expression in other breast cancer subtypes is only 30%, making FR an appealing target for breast cancer immunotherapy.^170^, ^171^ As a result, after infusion into a murine xenograft model of human TNBC, FR‐specific CAR T cells with an intracellular CD27 co‐stimulatory signalling region elicited significant tumour regression.^171^ Furthermore, fourth‐generation FR‐CAR T cells carrying extracellular FR‐specific scFv and three intracellular costimulatory domains (CD28, 4‐1BB, and CD27) linked to CD3 demonstrated specific cytotoxicity. They could eliminate about 88.7%of the target cells when cocultured with the FR‐expressing MDA‐MB‐231 BC cell line, whereas this effective antitumor activity was not observed against the FRα‐negative cell line.^172^

Mucin 1 (MUC1) is another intriguing target antigen that is overexpressed in various primary malignancies, including lung, gastric, breast, ovarian, colon, pancreatic, and prostate cancer. Furthermore, the glycosylated tumour form of MUC1 was expressed in 95% of all TNBC cells, linked to tumour invasiveness and metastatic potential in TNBC.^173^, ^174^ MUC1‐specific CARs with a combined CD28/OX40/CD3 endodomain and the HMFG2 scFv promoted proliferation and proinflammatory cytokine production in MUC1‐CAR T. Furthermore, these CAR T cells delayed tumour growth in a xenograft model bearing MDA‐MB‐435 tumour cells after a single dose of CAR‐engineered T cells was administered intraperitoneal (IP).^174^ In addition, Baigain et al. found that transgenic T cells co‐expressed with the rabbit recombinant monoclonal MUC1 antibody (HMFG2) scFv and 4/7ICR (an inverted cytokine receptor) could selectively expand and provide robust and prolonged cytotoxic activity in MUC1+ MDA MB 468 cell lines and IL4‐producing MDA MB 468 tumour‐bearing animals in vivo. These findings highlight the importance of presenting transgenic T cells with a combination of signals that characterize physiological TCR signalling – [activation (signal 1), co‐stimulation (signal 2), and cytokine support (signal 3)] – to ensure in vivo durability and memory constitution.^164^

Another study also indicated that establishing a CAR T‐cell specific for the cell‐surface protein c‐Met, which is present in 50% of TNBC, abrogated tumour development in c‐Met expressing tumour xenograft immune‐incompetent mice.^175^ Moreover, intertumoral injections of c‐Met CAR mRNA electroporated T cells into patients with metastatic breast cancer brought about wide tumour necrotic zones mainly achieved by cytolytic function mediated by mRNA c‐Met‐CAR T cells at the injection site; a lack of c‐Met immunoreactivity, and the recruitment of macrophages into intratumor areas, causing an inflammatory response within tumours. Thus, injection of mRNA c‐Met‐CAR T cells was well‐tolerated, and treating metastatic TNBCs with c‐Met‐CAR T cells was safe and practicable.^175^

Another target antigen is the ErbB receptor family, consisting of four transmembrane proteins: epidermal growth factor receptor (EGFR or ErbB‐1) and ErbB‐2 (HER2 or neu) ErbB‐3, and ErbB‐4. On nonmalignant cells, they are expressed but at modest levels. Overexpression of the ErbB family is prevalent in the pathogenesis of a variety of cancers, including head and neck, breast, lung, gastrointestinal tract, prostate, gynecologic tract, and pancreas, and improper ErbB signalling increases resistance to traditional therapeutic modalities such as hormonal agents, chemotherapy, and radiotherapy.^176^

Approximately 20% to 30% of all breast cancers overexpress HER2, impacting relapse rates and survival.^177^ Recently, designing second‐generation HER2‐specific CAR T cells with 4‐1BB intracellular costimulatory signalling domains and administering them intraventricularly demonstrated a robust in vivo antitumour impact in xenograft mouse models of breast metastatic brain tumours.^178^ Furthermore, isolation of CD3+ T‐cells from human PBMC followed by genetic modification with CAR specific for the HER2 was demonstrated to induce apoptosis in the HER2 overexpressing human breast cancer cell line, SKBR3, compared to non‐transduced T‐cells.^179^ In this light, Davies and coworkers generated and transduced to T cells a CAR structure consisting of a promiscuous ErbB ligand, T1E, coupled to a CD28+CD3 endodomain.^176^ T1E28z + T cells identified and destroyed breast cancer cell lines with a wide range of ErbB receptor profiles. Furthermore, treated mice showed substantial anticancer efficacy.^176^

Moreover, other studies found that CARs with 4‐1BB/CD3 endodomains fused to the extracellular domain of heregulin‐1 (HRG1), a natural ligand for HER3/HER4, boosted the killing potential of CAR‐T cells against HER3‐overexpressing SK‐BR‐3 and BT‐474 breast cancer cell lines. HRG1‐based CAR‐T cells also enticed intense antitumour activity in a xenograft model with SK‐BR‐3 tumour cells.^180^

Interestingly, creating EGFR‐specific CAR‐T cells targeting EGFR (HER1) in high‐EGFR expressing TNBC cells (HS578T, MDA‐MB‐468, MDA‐MB‐231) in vitro triggered cell death. Also, these CAR‐T cells inhibited the development of patient‐derived xenograft (PDX) TNBC tumours in mice.^181^ In other research, the third generation of CAR targeting EGFR incorporating intracellular signalling domains from CD28, 4‐1BB, and CD3 was created and transfected using a lentiviral vector into primary T cells. In EGFR‐expressing TNBC cells (MDA‐MB‐231 and MDA‐MB‐468 cell lines), these EGFR CAR‐T cells demonstrated compelling and specific toxicity in a dose‐ and time‐dependent manner. Furthermore, cytokine levels, such as TNF, IL‐2, and IFN‐γ, were more significant in EGFR CART cells following incubation with MDA‐MB‐231 or MDA‐MB‐468 cells than in non‐transfected T cells. This significant anticancer efficacy was confirmed in vivo in a xenograft mice model.^182^

Human endogenous retrovirus‐K (HERV‐K) is another potent antigen that can be targeted for CAR‐T treatment. It is produced in malignant BC cells at high levels but is missing in other normal tissues and nonmalignant cells. Engineering CAR T‐cells targeting the HERV‐K envelope protein (K‐CAR T cells) was able to suppress the proliferation of breast cancer cell lines while increasing cytokine release in the culture medium of BC cells treated with K‐CARs. In a xenograft model of MDA‐MB‐231 or MDA‐MB‐435, K‐CAR T cells successfully suppressed tumour development and metastasis to other organs.^183^

Tumour endothelial marker 8 (TEM8) is an integrin‐like cell surface marker that is overexpressed on the endothelium of various solid cancers and is thought to have a role in vascular cell migration and tumour invasion. Recent evidence has suggested that TEM8 expression, as a possible marker, in TNBC tumour‐associated vessels, TNBC tumour cells, and especially breast cancer stem‐like cells, play a crucial role in TNBC pathogenesis and invasion.^184^, ^185^, ^186^ In 2017, Byrd et al. found that engineering of primary human T cells by 2nd generation (CD28.CD3‐) and 3rd generation (CD28.41BB.CD3‐) TEM8‐specific CAR molecules derived from the scFv of the TEM8 antibody could effectively kill TEM8+ TNBC tumour cell lines such as Hs578T, MDA‐MB‐231, MDA‐MB‐436 and MDA‐MB‐468 and human breast tumour endothelial line HC 6020 and murine tumour endothelial cell lines 2H11 and bEND.3.^187^ Moreover, transferring TEM8 CAR T cells into MDAMB‐468 tumour‐bearing mice caused robust tumour regression by killing TEM8+ TNBC tumour cells, targeting the tumour endothelium and decreasing tumour vascularization. This discovery suggested that immunotherapeutic targeting of TEM8 might be used as a technique for CAR‐T cell treatment of TNBC.^187^ Besides that, targeting another marker antigen associated with a breast cancer stem‐like cell (BCSC) phenotype recognized as the disialoganglioside GD2 and producing novel anti‐GD2 CARs and expressing them on activated CD4 and CD8 positive T cells significantly lysed GD2‐positive breast cancer cells in vitro as well as halted local tumour progression and completely prevented lung metastasis in an orthotopic xenograft model of TNBC (MDA‐MB‐231 cell bearing rodent) in vivo.^188^ Another study targets AXL, a receptor tyrosine kinase (RTK) that overexpresses in various tumours, particularly breast cancer, with AXL‐CAR‐T cells containing 3rd generation (CD28.41BB.CD3‐) of AXL‐specific CAR led to significant cytolytic activity and cytokine secretion against AXL positive cells MDA‐MB‐231 in vitro. Significantly, intravenous injection of AXL‐CAR‐T cells into the mouse model of TNBC MDA‐MB‐231 xenografts inhibited tumour development. AXL‐CAR‐T cells have the potential to be a viable treatment method in AXL‐positive breast cancer.^189^ Moreover, engineered T cells with a CAR composed of the extracellular domain of NKG2D and signalling through CD3 and CD27/4‐1BB targeting NKG2D ligands (NKG2DLs) elicited compelling antitumor efficacy against TNBCs in vitro and in vivo in MDA‐MB‐231 xenograft mice.^190^

Mesothelin (Meso) is a cell‐surface protein that plays a vital role in tumour development, apoptosis resistance, and metastatic progression and is overexpressed in 67%of TNBC.^175^ As a result, it might be a promising target for cancer CAR‐T treatment. Li et al. revealed that designing Meso‐CAR‐T with co‐stimulation domains including CD28, 4‐1BB, and CD3 detected potent cytotoxicity in breast cancer MDA‐MB‐231‐Luc and MCF‐7‐Luc cell lines by releasing cytokines, perforin, and granzyme B as well as inhibition of tumour growth at a late stage in mice bearing MDA‐MB‐231 TNBC xenografts.^191^ Furthermore, combining Meso‐CAR‐T cells with rAd.sT, a TGF signalling‐targeted oncolytic adenovirus (Ad) potentiated Meso‐CAR‐T cell therapeutic effects and demonstrated considerably more spectacular antitumor activity against breast cancer and metastasis formation.^191^

## 10 OVERVIEW OF RECENT STUDIES BASED ON CAR‐NK CELLS IN BREAST CANCER

NK cells are cytotoxic lymphocytes that engage in the innate immune system. They play a crucial role in cancer immunosurveillance and have the potential to be a superb effector cell type for adoptive CAR‐based cancer immunotherapy. CAR‐redirected NK cells can be used as universal CAR‐based cells against tumour cells expressing particular antigens without the need for previous sensitization or HLA matching.^16^ These characteristics of CAR‐redirected NK cells provide a new avenue of attack in the fight against cancer (Table 3).

**TABLE 3:** Overview of different antigen‐specific CAR NK‐cells preclinical studies for breast cancer

Target Ag	CAR design	CAR transduction	Key preclinical outcomes	Ref
HER2/neu	CD28/CD3ζ	Electroporation	Reduced progressive signals in HER2/neu‐positive breast cancers in tumour‐bearing mice by NK‐92 scFv(FRP5)‐zeta Cells (in vivo)	^192^
HER2/neu	CD28/CD3ζ	Lentiviral	HER2‐expressing MDA MB468 breast cancer cell lines were successfully lysed (in vitro) and anticancer efficacy was preserved in mouse models of orthotopic breast carcinoma xenografts (in vivo) by NK‐92 scFv(FRP5)‐zeta cells	^193^
HER2/neu	CD3ζ	Lentiviral	Inducing apoptosis and completely eliminating ErbB2‐expressing MDA‐MB453 SKBR3 breast carcinoma cell lines (in vitro) and inhibiting the in vivo growth of ErbB2‐expressing tumour cells by NK‐92 scFv(FRP5)‐zeta cells	^194^
HER2/neu	CD28/CD3ζ	Electroporation	Enhancing the cell death of the HER2‐expressing human breast cancer cell lines MDA‐MB‐ 453 and SKBr3 (in vitro) and reducing tumour size and lung metastasis of nude mice bearing established MDA‐MB‐453 cells (in vivo) by NK‐92 scFv(FRP5)‐zeta cells	^195^
HER2	CD3ζ	Lentiviral	Improving the targeting of immune cell therapy of tumours metastasized to the brain by NK‐92‐scFv(FRP5)‐zeta cell line	^196^
EGFR	CD28/41BB/CD3ζ	Lentiviral	Cytokine secretion and cytotoxic effects on HS578T, MDA‐MB‐468, and MDA‐MB‐231 TNBC cell lines expressing upregulated EGFR (in vitro) and reducing tumour size in xenografts	^197^
EGFR	CD28/CD3ζ	Lentiviral	EGFR‐CAR NK‐92 cells increased cytolytic effect and IFN‐γ production in breast cancer cell lines MDA‐MB‐231, MDA‐MB‐468, and MCF‐7(in vitro) and mitigated tumour growth in tumour‐bearing mice (in vivo)	^198^
L‐ICON1 (Tissue factor)	CD28/41BB/CD3ζ	Lentiviral	Eliminating TF‐positive MDA‐MB‐231 cells (in vitro) and inhibition of tumour development in xenografts (in vivo)	^25^
EpCAM	CD28/CD3ζ/ encoding IL‐15	Lentiviral	Specific lysis of EpCAM‐expressing breast carcinoma cell lines (in vitro) by NK‐92/31.28.z‐IL‐15 cells	^199^

*Note*: Many studies have been carried out that the use of different appropriate antigens, designing efficient CAR structure, and improving gene transduction methods to enhance the efficiency of CAR NK therapy in breast cancer, which is summarized in the table.

Intravenous injection of NK‐92‐scFv(FRP5)‐zeta cells expressing a CAR specific to the tumour‐associated (HER2/neu) antigen into the tail vein of mice bearing HER2/neu‐positive NIH 3 T3 breast cancers was shown to reduce progressive signals in HER2/neu‐positive breast cancers 12 and 24 hours after injection.^192^ Furthermore, HER2‐expressing MDA‐MB468 breast cancer cell lines were successfully lysed in vitro by genetically engineered NK‐92‐scFv(FRP5)‐zeta cells producing a humanized CAR based on HER2‐specific antibody FRP5 carrying CD28 and CD3 signalling domain. In addition, CAR‐NK92 cells instigated anticancer impacts in mouse models of orthotopic breast carcinoma xenografts. They decreased the number of pulmonary tumour nodules as pulmonary metastasis in a renal cell carcinoma (RCC) animal model.^193^ In this study, irradiation was used to prevent NK cell line replication as a potential safety strategy for clinical trials, but the NK cell line's anticancer efficacy was intact.^193^ These results were consistent with those of Uherek et al. and Liu et al., who developed HER2‐specific scFv expressing NK92 cells and showed that HER2‐CAR NK92 cells induced a considerable increase in destruction and growth inhibition HER2‐expressing cancer cells in vitro and in vivo.^194^, ^195^ Interestingly, these findings were in line with a later study that found that intravenously injecting NK‐92‐scFv (FRP5)‐zeta cells expressing HER2‐specific CAR might reduce tumour volume in a xenograft HER2‐representing breast metastasis model in‐vivo.

Alkine et al. also discovered for the first time that ultrasound (FUS) was capable of augmenting the capacity of delivering targeted CAR‐NK‐92 cells to the brain using a xenograft model of metastatic breast cancer and was also capable of improving the targeting of immune cell therapy of tumours metastasized to the brain.^196^ Moreover, other studies have been undertaken to establish that EGFR‐CAR‐NK cells may be utilized to treat patients with TNBC who have elevated EGFR expression. Liu et al. generated EGFR‐CAR NK cells by transducing lentiviral vectors containing third‐generation CAR (Lenti‐EF1a‐scFv‐3rd‐CAR) with intracellular costimulatory domains (CD28, 4‐1BB) linked to CD3. They discovered that EGFR‐CAR NK cells have cytotoxic and antitumour effects on HS578T, MDA‐MB‐468, and MDA‐MB‐231 TNBC cell lines, exhibiting upregulated EGFR expression in‐vitro. When EGFRCAR‐NK cells were cocultured with TNBC cells with upregulated EGFR expression, they secreted more IFN‐γ, granzyme B and perforin than when cocultured with them a non‐TNBC cell line. In addition, the average weight and volume (size) of xenograft TNBC tumours in mouse models treated with EGFR‐CAR‐ NK cells were significantly reduced, indicating that the cells inhibited tumour growth in vivo.^197^ Another study combined EGFR‐CAR NK‐92 cells with oncolytic herpes simplex virus (oHSV) increased cytolytic effect and IFN‐γ production when co‐cultured with breast cancer cell lines MDA‐MB‐231, MDA‐MB‐468, and MCF‐7 in vitro compared to monotherapies. Moreover, compared to monotherapies, this combination demonstrated more effective cytolytic ability in MDA‐MB‐231 tumour cells and longer lifespan of mice intracranially pre‐inoculated with EGFR‐expressing MDA‐MB‐231 cells.^198^

Tissue factor (TF) is a new surface antigen expressed in 50%–85% of TNBC patients. In orthotopic mice models of TNBC cell line–derived and PDX, genetically altering NK cells to express TF‐targeting CAR followed by CD28, 4‐1BB, and CD3 zeta indicated therapeutic effectiveness and safety TF‐CAR‐NK cells (CDX and PDX). The results revealed that TF‐CAR‐NK cells significantly inhibited tumour development and reduced tumour weight. However, mouse body weights did not change considerably following in vivo treatment with TF‐CAR‐NK cells. In addition, in vitro data demonstrated effective direct cytotoxicity against TF‐positive MDA‐MB‐231 cells, suggesting that their efficacy may be enhanced when TF‐CAR‐NK cells are combined with second‐generation TF‐targeting therapeutic antibody‐like immunoconjugates, such as L‐ICON1.^25^

Moreover, Sahm et al. observed transduction of effector NK‐92 cells with a lentiviral vector encoding IL‐15 and a second‐generation CAR targeting epithelial cell adhesion molecule (EpCAM), a type I transmembrane glycoprotein identified as a TAA, could increase in vitro in the absence of any exogenous cytokines. The engineered CAR‐T cells displayed strong and specific cell‐killing actions against EpCAM‐expressing breast carcinoma cells, resistant to the natural cytotoxicity produced by unmodified NK cells.^199^

Studies on CAR‐NK cells collectively deliver a few vital signs for upcoming development. NK‐tailored CAR structure appears to be essential for maximizing NK cell cytotoxicity. Also, optimizing a procedure for the expansion and activation of harvested NK cells is required to attain a homogeneous population of clinically substantial counts of memory‐like, unexhausted NK cells. Additionally, the utility of CAR‐NK cells in the treatment of breast cancer may need further modifications of the NK cells beyond CAR transduction to increase trafficking and desensitize them to the immunosuppressive TME.^200^

## 11 CLINICAL TRIALS FOR CAR‐T CELL THERAPY IN BREAST CANCER

Numerous researches have been carried out at the preclinical in vitro and in vivo levels of treatment with CAR‐T cells in breast cancer, which has progressed to first‐in‐human studies, as shown in Table 4. A phase I trial was initiated to assess the safety and optimal dose of intraventricularly administered autologous HER2‐targeted chimeric antigen receptor (HER2‐CAR) T cells in patients with brain and/or leptomeningeal metastases from HER2 positive cancers (NCT03696030). Likewise, a phase I clinical trial was conducted to assess the safety and feasibility of injecting c‐Met‐CAR‐T cells intratumorally into patients with metastatic breast cancer (NCT01837602). This trial demonstrated that treatment with c‐Met‐CAR T cells was well‐tolerated by patients and elicited extensive tumour necrosis and was observed at the injection site inflammatory response within tumours, with no evidence of side effects greater than grade 1. So far, most studies have been carried out on MUC1‐CAR‐T cell therapy in clinical trials. The safety and effectiveness of autologous MUC1‐CAR‐T cells for patients with advanced refractory TNBC were investigated in Phase I/II study (NCT02587689).

**TABLE 4:** Clinical trials based on CAR T cell therapy in breast cancers registered in clinicaltrials.gov (February 2022)

Phase	Participant No	Status	Location	Type	Target antigen	NCT number
1	39	Recruiting	USA	Interventional	HER2	NCT03696030
1/2	na	Withdrawn	China	Interventional	HER2	NCT02547961
1/2	20	Unknown	China	Interventional	Mucin1	NCT02587689
1	220	Recruiting	USA	Interventional	HER2	NCT04650451
1	69	Active, not recruiting	USA	Interventional	Mucin1	NCT04020575
1	75	Unknown	China	Interventional	CEA	NCT02349724
1/2	na	Withdrawn	China	Interventional	HER2	NCT02713984
1	30	Recruiting	China	Interventional	EP‐CAM	NCT02915445
1	6	Completed	China	Interventional	c‐MET	NCT01837602
1	112	Recruiting	USA	Interventional	Mucin1	NCT04025216
1	45	Recruiting	USA	Interventional	HER2	NCT03740256
1	10	Unknown	Malaysia	Interventional	NKG2D ligand	NCT04107142
Early 1	77	Terminated	USA	Interventional	c‐MET	NCT03060356
1	18	Active, not recruiting	USA	Interventional	CEA	NCT03682744
1/2	2	Suspended	USA	Interventional	CD70	NCT02830724
1/2	113	Active, not recruiting	USA	Interventional	Mesothelin	NCT02414269
1	60	Recruiting	USA	Interventional	ROR1	NCT02706392
1	94	Recruiting	USA	Interventional	GD2	NCT03635632

*Note*: In the present table, we summarized registered studies evaluating the safety and efficacy of CAR‐T cell therapy in patients with breast cancer.

Moreover, Minerva Biotechnologies Corporation is developing adoptively transferred autologous T cells genetically modified to express a CAR that targets a cleaved form of MUC1 antigen, which is being evaluated against patients with metastatic breast cancer in phase I clinical trial (NCT04020575). Besides, an open‐label phase I trial was started to assess the preliminary efficacy of TnMUC1‐CAR‐T cells administered intravenously to patients with advanced TnMUC1‐positive breast cancer tumours (NCT04025216). Other CAR‐T cell targets under investigation in the clinical trial for relapsed or refractory breast cancer include NKG2D ligands and EpCAM. A phase I study was designed to assess the safety and tolerability of intravenous infusions of allogeneic NKG2DL‐targeting Chimeric Antigen Receptor‐grafted Gamma Delta T Cells at three target dose levels ranging from 3x108 to 3x109 per infusion (NCT04107142). In addition, a phase I study is recruiting patients with breast cancer to evaluate the safety of autologous engineered T cells armed with chimeric antigen receptor (CAR‐T) recognizing EpCAM. The number of CAR‐T cell therapies in clinical trials is growing, paving the way for an advanced immunotherapy course in breast cancer.

## 12 CONCLUSION AND FUTURE DIRECTION

There is an unmet therapeutic necessity to develop effective therapies for breast cancer patients with a high risk of recurrence and metastasis and a low survival rate. Immunotherapeutic techniques based on CAR‐redirected immune cells have evolved, with the potential to redirect immune cells such as T and NK cells to suppress malignancies,^201^, ^202^, ^203^ with various conducted or ongoing clinical trials. Recent studies have concentrated on selecting the most appropriate therapeutic target, improving the CAR structure for ideal immune cell functions, conducting extensive preclinical and clinical trials, developing mechanisms to overcome barriers that lead to defects in CAR‐immune cell safety and efficiency, and improving the specificity, competence, and resistance of these engineered cells.^24^ Several obstacles related to CAR‐redirected immune cell treatment in breast cancer must be considered to make this strategy safer and more effective. One of the critical issues is identifying optimal antigenic targets in breast cancer with a deregulated expression on both primary tumour cells and cells resident in the TME (e.g., MDSCs, TAMs, CAFs, Tregs) overcoming tumour escape.^204^, ^205^, ^206^ This topic describes how to engineer T and NK cells to target multiple markers expressed on breast cancer cells and stromal cells and use factors that can upregulate the expression of specific antigenic targets on the cell surface and boost sensitivity to CAR‐based cells.

Furthermore, incorporating cytokine receptors such as IL‐15 or IL‐7 receptors or proinflammatory cytokines into CAR‐based immune cell constructs can be used to reprogram the immunosuppressive TME and extend the survival of CAR‐T and CAR‐NK cells in the hostile TME of breast cancer.^207^, ^208^, ^209^ In addition, appropriate chemokine receptors may be integrated into modified cell structure to increase directed migration and boost CAR‐T and CAR‐NK cell infiltration to the tumour site.^210^, ^211^ Furthermore, numerous suicide genes, such as the inducible caspase‐9 (iC9)‐based suicide gene, can be used as safety switches in breast cancer engineered cell constructions, improving cell safety and reducing the risk for tumour/off‐target damage.^32^ Combining CAR‐based immune cell therapy with other therapeutic techniques is another effective option for treating breast cancer that can target many mechanisms simultaneously. Combining CAR‐T cell treatment with immune checkpoint inhibition (e.g., blocking antibodies of PD‐1/PD‐L1 and CTLA‐4) has been shown to impact tumour immunosuppressive forces and anticancer activity^212^, ^213^ significantly. Another idea is to use current developments in gene editing methods, such as CRISPR/Cas9‐based genetic changes, to maintain CAR‐NK cell safety and cytotoxicity function while posing no threat to normal tissues.^16^

## AUTHOR CONTRIBUTIONS

**Marzieh Nikoo:** Conceptualization (equal); writing – original draft (lead). **Mohammad Rudiansyah:** Conceptualization (equal); writing – review and editing (equal). **Dmitry Olegovich Bokov:** Conceptualization (equal); writing – review and editing (equal). **Nurlan T. Jainakbaev:** Conceptualization (equal); writing – review and editing (equal). **Wanich Suksatan:** Conceptualization (equal); writing – review and editing (equal). **Mohammad Javed Ansari:** Conceptualization (equal); writing – review and editing (equal). **Lakshmi Thangavelu:** Conceptualization (equal); writing – review and editing (equal). **Supat Chupradit:** Conceptualization (equal); writing – review and editing (equal). **Amir Zamani:** Conceptualization (equal). **Ali Adili:** Writing – review and editing (equal). **Navid Shomali:** Writing – review and editing (equal). **Morteza Akbari:** Supervision (lead).

## CONFLICT OF INTEREST

There is no conflict of interests.

## Data Availability

Not applicable.
